# Infusion of Bone Marrow Mononuclear Cells Reduces Lung Fibrosis but Not Inflammation in the Late Stages of Murine Silicosis

**DOI:** 10.1371/journal.pone.0109982

**Published:** 2014-10-09

**Authors:** Miquéias Lopes-Pacheco, Túlio G. Ventura, Helena D'Anunciação de Oliveira, Leonardo C. Monção-Ribeiro, Bianca Gutfilen, Sergio A. L. de Souza, Patrícia R. M. Rocco, Radovan Borojevic, Marcelo M. Morales, Christina M. Takiya

**Affiliations:** 1 Laboratory of Cellular Pathology, Institute of Biophysics Carlos Chagas Filho, Federal University of Rio de Janeiro, Rio de Janeiro, Brazil; 2 Laboratory of Cellular and Molecular Physiology, Institute of Biophysics Carlos Chagas Filho, Federal University of Rio de Janeiro, Rio de Janeiro, Brazil; 3 Laboratory of Pulmonary Investigation, Institute of Biophysics Carlos Chagas Filho, Federal University of Rio de Janeiro, Rio de Janeiro, Brazil; 4 Department of Radiology, School of Medicine, Federal University of Rio de Janeiro, Rio de Janeiro, Brazil; 5 Laboratory of Cellular Proliferation and Differentiation, Biomedical Science Institute, Federal University of Rio de Janeiro, Rio de Janeiro, Brazil; University of California, Riverside, United States of America

## Abstract

We hypothesized that infusion of bone marrow mononuclear cells (BMMCs) in the late stages of silica-induced damage would reduce the remodelling process in a murine model of silicosis. C57BL/6 mice were assigned to 2 groups. In the SIL group, mice were instilled with a silica particle suspension intratracheally. Control (C) mice received saline under the same protocol. On the 40th day, some of the animals from both groups were killed. The others were treated with either saline or BMMCs (1×10^6^cells) intravenously (C+BMMC and SIL+BMMC), and the mice were killed 70 days after the start of the protocol. In the mice in the SIL+BMMC group, collagen deposition, the presence of silica particles inside nodules, the presence of macrophages and cells reactive for inducible nitric oxide synthase were reduced. Lung parameters also improved. Beyond that, the total and differential cellularity of bronchoalveolar lavage fluid, immunoexpression of transforming growth factor-β, the number of T regulatory cells and apoptosis were increased. However, the presence of male donor cells in lung tissue was not observed using GFP+ cells (40d) or Y chromosome DNA (70d). Therefore, BMMC therapy in the late stages of experimental silicosis improved lung function by diminishing fibrosis but inflammatory cells persisted, which could be related to expansion of T regulatory cells, responsible for the beneficial effects of cell therapy.

## Introduction

Silicosis is an occupational lung disease resulting from chronic inhalation of dust containing silica dioxide. It is characterized by persistent inflammation, fibroblast proliferation and excessive collagen deposition, resulting in interstitial fibrosis [1]. During the development of silicosis, contact between alveolar macrophages and silica drives the subsequent steps. The uptake of silica particles by macrophages triggers the production of reactive oxygen species (ROS) via the oxidative stress pathway, which in turn contributes to pulmonary damage and macrophage death by apoptosis [2], [3]. Sustained ROS generation perpetuates the continuum of phagocytosis, cell death, inflammatory cell recruitment and silica deposition, and is responsible for progressive and irreversible lung injury [4], [5]. The fibrosis and the inflammatory reaction inside the alveolar spaces lead to respiratory failure due to a reduction in the area of gas exchange and impairment of lung function. As yet, no curative treatment exists for silicosis. Clinical management is directed at controlling symptoms and preventing complications [6].

Therefore, transplantation of stem cells obtained from several sources has been proposed. In this context, an increasing number of articles have demonstrated the efficacy of either systemic or intratracheal administration of bone marrow cells in several lung injury animal models. This includes mouse models of acute lung injury and fibrosis [7], [8], sepsis [9], [10], ischemia/reperfusion injury [11], asthma [12], chronic obstructive pulmonary disease [13], [14], and other pulmonary diseases [15], [16]. It has been shown that bone marrow-derived cells are capable of promoting re-epithelization of lung parenchyma, modulating immune responses, and decreasing fibrosis [15], [17]. However, few studies have been done in the setting of a chronic persistent inflammatory and fibrotic condition such as silicosis. Maron-Gutierrez et al. [18] reported that bone marrow mononuclear cells (BMMCs) had a preventive effect when infused 1 h after the introduction of silica, with improvement in lung function, inflammation, and fibrosis 15 days after the start of the protocol. However, these effects were only partially reversed in a longer follow-up (60 days) in a protocol with infusion of bone marrow-derived cells by the intratracheal route in animals with a 15-day silica-induced injury [19]. Therefore, we asked if treatment with BMMCs in the chronic stages of murine silicosis could also have beneficial effects on lung function and structure.

## Materials and Methods

This study was approved by the Ethics Committee of the Carlos Chagas Filho Biophysics Institute, Health Sciences Centre, Federal University of Rio de Janeiro (CEUA-CCS-019). All animals received humane care in compliance with the “Principles of Laboratory Animal Care” formulated by the National Society for Medical Research and the “Guide for the Care and Use of Laboratory Animals” prepared by the National Academy of Sciences, USA.

### Experimental protocol

A total of 133 male and 24 female mice were used in this experiment. Ten-week-old male C57BL/6 mice (20–25 g) were randomly divided into groups: animals instilled intratracheally either with 50 µL of sterile saline (C group) or with silica suspension (particle size: 80% between 1 and 5 µm, 10 mg/50 µL of saline; Sigma-Aldrich, St. Louis, MO, USA) (SIL group). On the 40th day, some of the animals from both groups were killed (C40d and SIL40d). The others were treated either with saline (50 µL) (C70d and SIL70d) or BMMCs (1×10^6^cells/50 µL) via the tail vein (C+BMMC and SIL+BMMC), and the mice were then killed 70 days after the start of the protocol. The experiments comprised the following groups: C40d, C70d, C+BMMC, SIL40d, SIL70d, and SIL+BMMC.

Another group was prepared for the analysis of the presence of BMMCs in mice injected with silica. BMMCs from male mice (1×10^6^ cells/50 µL of saline) were injected intravenously into normal female animals (C+BMMC) or in female SIL mice (SIL+BMMC). The animals were killed 30 days later. Lungs were collected for detection of Y chromosome.

### Extraction of BMMCs

Bone marrow was harvested from the femur and tibia of 10-week-old male C57BL/6 mice (20–25 g) and injected on the same day. Cells were aspirated by flushing the bone marrow cavity with Dulbecco's modified Eagle's medium (Life Technologies, Grand Island, NY, USA). After a homogeneous cell suspension was achieved, cells were centrifuged (400× for 10 min), re-suspended and added to Ficoll-Paque™ Plus (Amersham Biosciences, USA), and again centrifuged and re-suspended in phosphate-buffered saline. Cells were counted in a Neubauer chamber with Trypan Blue for evaluation of viability.

### Biodistribution of BMMCs label with 99m Technetium (Tc)

BMMCs were labeled with 99mTc following protocols described previously [20], [21]. Briefly, 500 µL of sterile SnCl_2_ solution was added to the cell suspension and the mixture was incubated at room temperature for 10 min. Then, 5mCi of 99mTC was added and the incubation continued for another 10 min. After centrifugation (500×*g* for 5 min), the supernatant was removed and the cells were washed three times with NaCl 0.9%. Viability of the labeled cells was assessed by the trypan blue exclusion test and was estimated to be greater than 93% in all cases. Labeling efficiency (%) was calculated by the activity in the pellet divided by the sum of radioactivity in the pellet plus supernatant, and was estimated to be greater than 90% in all cases. 1×10^6^ 99mTc-BMMCs were injected through the tail vein immediately after labelling. Whole-body scintigraphies were performed in these animals for qualitative biodistribution in a dedicated small animal SPECT/CT camera (Triumph, Gamma-medica ideas, Canada) equipped with high-resolution collimator and diagnostic CT for 2, 4 and 24 hours after 99mTc-BMMCs administration.

### Detection of Y chromosome DNA

Quantification of murine Y chromosome in lung tissue was achieved as previously described [18] using a quantitative real-time polymerase chain reaction at day 70. The following polymerase chain reaction primers were used: SRY forward: 5′-TCA TCG GAG GGC TAA AGT G-3′ and reverse: 5′-CAA CCT TCT GCA GTG GGA C-3′; GAPDH forward: 5′-CCA CCA ACT GCT TAG CCC-3′ and reverse: 5′-GAC ACC TAC AAA GAA GGG TCC A-3′.

### Expression of Green Fluorescent Protein (GFP)

The relative levels of expression of bone marrow derived mononuclear GFP+ cells in lung parenchyma were achieved by quantitative real-time RT-PCR. Left lung were cut, collected in cryotubes, quick-frozen by immersion in liquid nitrogen, and stored at −80°C. Total RNA was prepared using an RNeasy Plus Mini Kit according to the manufacturer's recommendations (Qiagen, Valencia, CA). RNA was quantified in a nanodrop spectrophotometer. First strand cDNA was synthesised from total RNA using GoScript™ Reverse Transcription System (Promega, Madison, USA). Relative mRNA levels were measured with a SYBR green detection system (GoTaq qPCR Master Mix; Promega, Madison, USA) in Eppendorf Mastercycler ep realplex thermal cyclers (Eppendorf, Hamburg, Germany). The following PCR primers were used: eGFP forward: 5′-CCA CAT GAA GCA GCA GGA CTT-3′ and reverse: 5′-GGT GCG CTC CTG GAC GTA-3′; 36B4 forward: 5′-CAA CCC AGC TCT GGA GAA AC-3′ and reverse: 5′-GTT CTG AGC TGG CAC AGT GA-3′.

### Mechanical parameters

On the 40th or 70th day after the start of the protocol, the mice were sedated (diazepam, 1 mg/kg i.p.), anaesthetized (thiopental sodium, 20 mg/kg i.p.), tracheotomized, paralyzed (vecuronium bromide, 0.005 mg/kg i.v.), and ventilated with a constant flow ventilator (Samay VR15; Universidad de la Republica, Montevideo, Uruguay) with the following parameters: tidal volume (V_T_), 0.2 mL; fraction of inspired oxygen, 0.21; and frequency, 100 breaths/min. The anterior chest wall was surgically removed and a positive end-expiratory pressure of 2 cmH_2_O was applied. Lung mechanics were computed 12 times per animal.

Resistance (ΔP1,l) and viscoelastic (ΔP2,l) pressures and lung static elastance (Est,l) were measured by the end-inflation occlusion method [22]. All data were analysed using ANADAT data analysis software (RHT-InfoData, Inc., Montreal, Quebec, Canada).

### Bronchoalveolar lavage fluid

Lungs were rinsed twice via a tracheal tube with phosphate-buffered saline solution (1 mL/wash) containing EDTA (10 mN). Total leukocyte numbers were measured in Neubauer chambers under light microscopy after diluting the samples of bronchoalveolar lavage fluid (BALF) in Türk solution.

Differential cell counts were performed on cytospin smears stained by the May-Grünwald-Giemsa method.

### Myeloperoxidase (MPO) and lactate dehydrogenase (LDH) activity

After collection of BALF, individual lungs (from the different groups) were collected and cut into pieces of equal weight (100 mg). To measure the MPO activity, lung tissue was suspended in 1 mL of buffer containing 0.5% hexadecyltrimethylammonium bromide in 50 mM phosphate buffer (pH 6.0) and sonicated. Homogenates were cleared by centrifugation at 10,000×*g* for 15 min at 4°C, and the supernatants (50 µL) were added to 50 µL of substrate solution containing 5 mM *o*-phenylene diamine (Sigma-Aldrich) in 10 mM citrate buffer, pH 5.0, and 8.8 mM H_2_O_2_. The reaction was stopped after 15 min with 50 µL of 4 N H_2_SO_4_, and the absorbance was read at 492 nm. Results shown are from all the mice in each group, and each point represents the mean of triplicate readings.

Lung tissue lactate dehydrogenase (LDH) activity was determined using a commercial kit (Gold Analisa Diagnóstica, São Paulo, Brazil). Protein concentration was determined according to the Griess method. The assay is based on reduction of NAD by LDH. The resulting reduced NAD (NADH) is utilized in the stoichiometric conversion of a tetrazolium dye. The resulting coloured compound is measured spectrophotometrically at 340 nm. One unit causes the oxidation of one micromole of NADH per minute at 25°C and pH 7.3, under the specified conditions. LDH activity was presented as millimoles of NADH oxidized per milligram of protein.

### Lung histology

Lungs were fixed at end expiration with 10% buffered formaldehyde solution and embedded in paraffin. Sections (4-µm thick) were cut and stained with haematoxylin–eosin (H-E) or a modified Sirius Red to quantify collagen.

### Histomorphometry and stereology of silicotic nodules

Thirty photomicrographs of H&E-stained sections were used to obtain the cellularity of silicotic nodules and for stereological analysis of the silicotic nodules to obtain the area and volumetric density [23]. Quantification of the area of the nodules was done by outlining the structures and the results are shown in square micrometres (µm^2^). For the volumetric density, we used the formula: (P_p_/P_t_ = A_p_/A_t_ = L_p_/L_t_ = V_p_/V_t_), where P_p_ are partial points, L_p_ are partial lines, P_t_ are total points, L_t_ are total lines, A_p_ is partial area, V_p_ is partial volume, A_t_ is the total area, and V_t_ is the total volume. A grid with 36 test points was projected onto the image and the points that touched the nodules were counted. The volumetric density of the nodules (Vv[nodules]) was estimated by the formula: Vv(nodules)  =  P_P_(nodules)/P_T_.

Results for cellularity in the nodules are expressed as the mean±standard deviation of the amount of cells per field. After obtaining the area of the nodules (µm^2^), they were separated into 2 ranges: those with <100,000 or>100,000 µm^2^. The frequency was obtained and presented as the amount of nodules per field.

### Lung immunohistochemistry/histochemistry and histomorphometry

Immunostaining was performed in paraffin sections using the peroxidase method and the chromogen substrate diaminobenzidine (DAB liquid, catalogue no. K3468, Dakocytomation, Carpinteria, CA, USA). Antibodies were detected using a secondary antibody labelled with peroxidase from Nichirei Biosciences, Japan (Histofine mouse MAX PO and anti-rabbit) followed by DAB. Negative controls were incubated with rabbit non-immune sera or with the antibody diluent (catalogue no. S3022, Dakocytomation).

The following antibodies were used: transforming growth factor (TGF)-β, a rabbit polyclonal antibody against pan-transforming growth factor-β (1 µg/mL; catalogue no. AB-100NA, R&D Systems, USA), a rabbit polyclonal antibody against the enzyme nitric oxide synthase, inducible (iNOS) (1 µg/mL; catalogue no. RB-9242, LabVision, Fremont, CA, USA) and GFP rabbit IgG polyclonalantibody fraction (2 µg/mL; catalogue no A-11122, Life Technologies, Carlsbad, CA, USA).

The biotinylated lectin, Griffonia (Bandeiraea) simplicifolia lectin 1 (BSL-1) (10 µg/mL; Vector Laboratories, Burlingame, CA) was used for staining alveolar macrophages [18], [24]. Negative control slides were incubated with inhibiting sugar solution (200 mM galactose/200 mM *N*-acetylgalactosamine mixture). For detection of biotinylated lectin, paraffin sections were dewaxed and hydrated, and after inhibition of endogenous peroxidase and biotin (Streptavidin blocking kit, catalogue no. SP-2002, Vector Laboratories, Burlingame, CA, USA), the sections were incubated with streptavidin-peroxidase (2 µg/mL; catalogue no. SA-5004, Vector Laboratories), and revealed using DAB.

Thirty microscopic fields from alveolar septae or granulomatous nodules were randomly selected, avoiding vessels and bronchi, and high-quality images (2,048×1,536 pixel buffer) were captured after setting and calibrating the program, using the 40× objective lens. The images were analysed using Image Pro Plus 4.5.1 software (Media Cybernetics).

Cells reactive for iNOS inside nodules were quantified and expressed as the % of iNOS-reactive cells in the total inflammatory cells inside nodules. The area occupied by TGF-β either in alveolar septae or nodules was expressed as the % reactive area in the septae and nodules, separately, in the total area captured. BSL-1 results are expressed as the % of alveolar macrophages cells in the total of microscopic fields of alveolar septae or nodules.

### T regulatory cells (FoxP3)

In order to verify if T regulatory cells were present in silica-induced lung disease, we performed an immunohistochemical assay to detect this subpopulation. The anti-FoxP3 antibody (ab10563, AbCam, USA) was used.

### Silica crystals in nodules

Silica particles are small crystals and can be viewed by polarized light; they appear as granular birefringent particles. We used this property to quantify the amount of silica present in the lung tissue [25]. Twenty microscopic, randomly selected, non-coincident fields were captured using polarized light (40× objective lens) from sections stained with H&E. The birefringent material present inside nodules was quantified. The results are expressed as the % of silica in the microscopic fields.

### In situ apoptosis

For detection of apoptosis, we used the TUNEL (terminal deoxynucleotidyl transferase [TdT]-mediated dUTP nick-end labelling) method. The ApopTag peroxidase in situ apoptosis detection kit (Merck Millipore) was used according to the manufacturer's instructions. Quantification was performed in 20 random and non-coincident microscopic fields (40× objective lens). The results are expressed as the number of positive cells in the total microscopic fields.

### Collagen histomorphometry

For histomorphometry, we used an image analysis system composed of a light microscope (Eclipse E800, Nikon, Japan) coupled to a digital camera (Evolution Media Cybernetics Inc., Bethesda, MD), and a computer with the graphical interface software Q-Capture 2.95.0, version 2.0.5 (Silicon Graphic Inc, USA). High-quality images (2,048×1,536 pixel buffer) were captured after setting and calibrating the program, using the 40× objective lens. To measure the collagen content, 20 photomicrographs of both alveolar septae and silica-induced nodules were obtained, separately, from sections stained with Sirius Red. The results are expressed as the % of reactive tissue in the total area.

### Enzyme-linked immunosorbent assay

Levels of interleukin (IL)-10 and TGF-β were quantified in lung tissue by enzyme-linked immunosorbent assay according to the manufacturer's instructions (Duo Set, R&D Systems, Minneapolis, MN). The results are expressed as pg/mL.

### Statistical analysis

Statistical analyses were performed using GraphPad Prism 4.0 (GraphPad Software, San Diego, CA, USA). The normality of the data (Kolmogorov-Smirnov test with Lilliefors' correction) and the homogeneity of variances (Levene median test) were tested. Differences between the groups were assessed by Kruskal-Wallis or one-way analysis of variance followed by the Tukey test. Data are presented as the mean±SEM. In all tests, the significance level was set at 5%.

## Results

### Homing and engraftment of BMMCs

**Figure 1:**
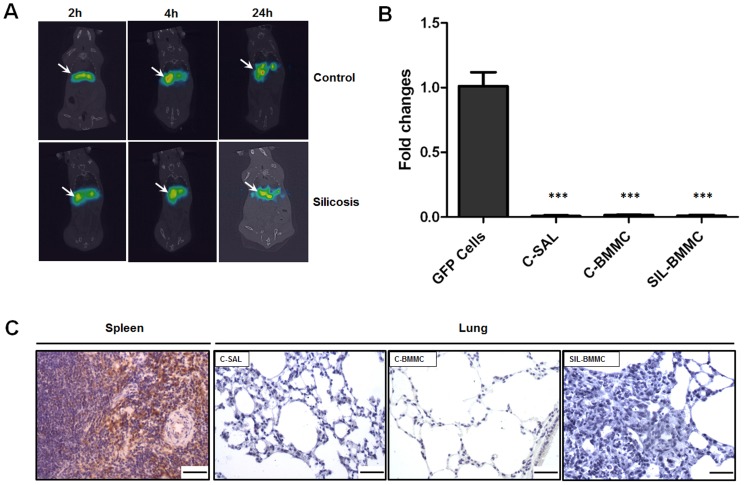
Biodistribution and homing of BMMCs. (**A**) Representative coronal whole-body SPECT/CT images of control and silicosis animals 2, 4 and 24 h after endovenous 99mTc-BMMCs shows liver uptake until 24 h. n = 2 animals per group. 99mTc-BMMCs were found predominantly in the liver (arrows). (**B**) Real-time PCR analysis of GFP mRNA expressions. Adipose-derived mesenchymal stem cells from GFP mice were used as positive control (GFP Cells). Control (C) and silica group (SIL) mice received saline or silica intratracheally. C and SIL animals were treated with BMMC from GFP mice (C-Cell and SIL–cell) or saline (C-SAL). Data are presented as mean ± SEM. n = 4 animals per group. ***Significantly different from C-GFP (P<0.001). (**C**) Photomicrographs of spleen and lung parenchyma after imunohistochemistry with GFP antibody. GFP positive cells were not observed in lung parenchyma of C+BMMC and SIL+BMMC animals 24 h after treatment with BMMC from GFP mice. Spleen of GFP male mice were used as positive control. Bars: 100 µm. n = 4 animals per group.

Biodistribution and homing of 99mTc-BMMCs was evaluated 2, 4 and 24 hours after infusion in C and SIL groups. No significant differences were observed among the time points and experimental groups (C+BMMC and SIL+BMMC) (Fig. 1A). Also, GFP+ cells were not detected in lung tissue in 24 h after BMMCs infection (Fig. 1B,C).

Y chromosome DNA was not detected in the lungs of SIL+BMMC or C+BMMC animals, 30 days after BMMCs administration (data not shown).

### Lung function

**Figure 2:**
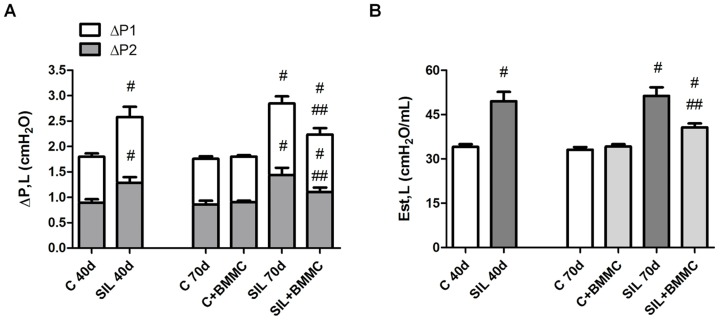
BMMC therapy improves lung function in SIL mice. (**A**) Resistance (ΔP1,l) and viscoelastic (ΔP2,l) pressures and (**B**) lung static elastance (Est,l). Mice in the control (C) and silica (SIL) groups received saline or silica intratracheally. C and SIL animals were treated with BMMCs (1×10^6^ cells i.v., C+BMMC and SIL+BMMC). Data are presented as the mean ± SEM. *n* = 7 animals per group. ^#^Significantly different from C. ^##^Significantly different from SIL40d and SIL70d.

The values for ΔP1,l, ΔP2,l and Est,l were similar for the mice in the C40d, C70d and C+BMMC groups. Mice in the SIL40d and SIL70d groups presented higher ΔP1,l, ΔP2,l and Est,l values compared with the mice in the C groups. Lung function was improved in the mice in the SIL+BMMC group (Fig. 2).

### Cellularity of BALF

**Figure 3:**
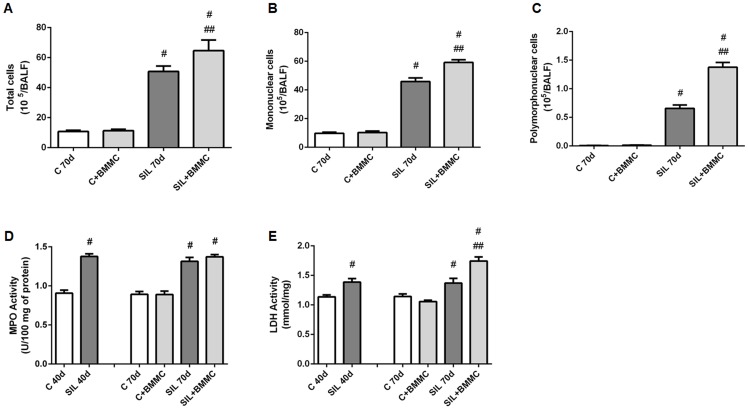
BMMC therapy increases cellularity in BALF, LDH activity and does not alter myeloperoxidase activity. Quantification of (**A**) total number of cells, (**B**) mononuclear cells and (**C**) polymorphonuclear cells in BALF. Quantification of (**D**) myeloperoxidase and (**E**) LDH activity. Mice in the control (C) and silica (SIL) groups received saline or silica intratracheally. C and SIL animals were treated with BMMCs (1×10^6^ cells i.v., C+BMMC and SIL+BMMC). Data are presented as the mean ± SEM. *n* = 7 animals per group. ^#^Significantly different from C. ^##^Significantly different from SIL40d and SIL70d.

The total cellularity in the samples of BALF was increased significantly in the mice in the SIL40d (data not shown) and SIL70d groups compared with the C40d (data not shown), C70d and C+BMMC animals; the number of mononuclear and polymorphonuclear cells was also increased. The SIL+BMMC animals had a significant increase in the number of inflammatory cells in the BALF (total and differential counts) compared with all groups (*P*<0.05) (Fig. 3A–C).

### Myeloperoxidase and LDH activity

Myeloperoxidase activity is an index of accumulating polymorphonuclear cells. Lung extracts obtained from SIL40d, SIL70d and SIL+BMMC animals showed an increase in myeloperoxidase activity (*P*<0.05) compared with animals in the C40d, C70d, and C+BMMC groups. There was no difference in the polymorphonuclear cell content between the SIL40d, SIL70d and SIL+BMMC animals (Fig. 3D).

Since LDH is an intracellular enzyme, any process causing injury to the cell will result in the release of LDH. This released LDH will cause an increase of LDH enzyme levels (which normally is very low). LDH activity was similar in the C40d, C70d and C+BMMC groups. SIL40d, SIL70d and SIL+BMMC animals showed increased LDH activity compared with C groups (*P*<0.05). The SIL+BMMC group presented more LDH activity than the SIL40d and SIL70d groups (Fig. 3E).

### Lung histology

**Figure 4:**
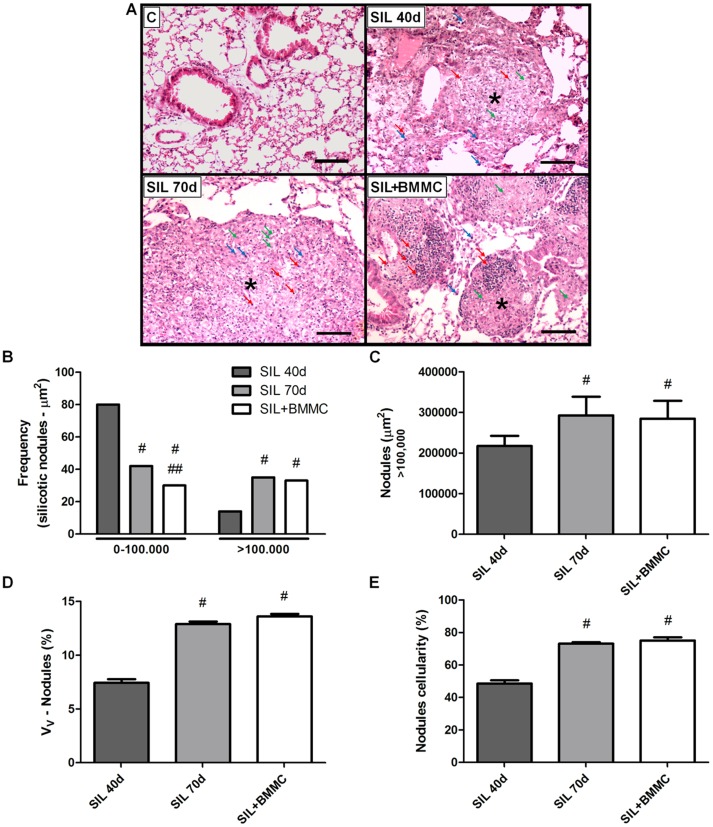
BMMC therapy does not reduce silicotic nodules. (**A**) Photomicrographs of lung parenchyma stained with H&E from animals in the C, SIL40d, SIL70d and SIL+BMMC groups. Note the presence of silicotic nodules (*) containing lymphocytes (red arrow), neutrophils (green arrow) and macrophages (blue arrow) in the SIL groups. Bars: 100 µm. Quantification of (**B**) frequency, (**C**) nodules (>100,000), (**D**) volume (Vv) and (**E**) cellularity of silicotic nodules in the SIL groups. Data are presented as the mean ± SEM. *n* = 7 animals per group. ^#^Significantly different from SIL40d. ^##^Significantly different from SIL70d.

After the instillation of silica, foamy macrophages, lymphocytes and neutrophil infiltrate became progressively more widespread in the lungs, forming nodular peribronchiolar infiltrates and multifocal fibrosing alveolitis. SIL40d and SIL70d animals presented various granulomatous nodules in lung parenchyma that were not seen in the C animals (Fig. 4A). Nodules in the SIL40d and SIL70d animals consisted mainly of epithelioid macrophages interspersed with some neutrophils, apoptotic cells, silica particles and lymphocytes. Some nodules contained proteinaceous material, cholesterol clefts and necrotic debris. In SIL+BMMCs animals, nodules appeared to be smaller than those seen in SIL40d and SIL70d animals. These nodules had less proteinaceous material, cholesterol clefts, and silica than those in SIL40d or SIL70d animals, and frequently contained aggregates of lymphocytes (Fig. 4A).

Stereological methods demonstrated that animals in the SIL40d group had a higher frequency of small nodules (<100.000 µm^2^) compared with animals in the SIL70d and SIL+BMMC groups (*P*<0.05). The nodules seen in SIL70d animals were various sizes but nodules were more frequent in the animals in the SIL70d group than in the SIL+BMMC group (*P*<0.05). A higher frequency of animals in the SIL70d and SIL+BMMC groups had nodules>100.000 µm^2^ compared with the SIL40d group. However, there were no differences in the frequency of this size of nodules between the groups (Fig. 4B). When comparing the size of the nodules based on the area, it was found that SIL70d and SIL+BMMC animals had nodules with larger area (*P*<0.05) compared with SIL40d animals. However, there were no differences in the area of nodules between both groups (*P*<0.05) (Fig.4C). The volumetric density (Vv) of silica nodules was significantly higher in SIL70d and SIL+BMMC animals compared with SIL40d animals (*P*<0.05) but the increased volumetric density was not different between the SIL70d and SIL+BMMC groups (Fig. 4D).

Quantification of the cellularity of silica nodules showed that animals in the SIL70d and SIL+BMMC groups had a higher quantity of inflammatory cells than SIL40d animals (*P*<0.05) but the increase in the number of cells inside nodules was similar in the SIL70d and SIL+BMMC animals (*P*<0.05) (Fig. 4E).

**Figure 5:**
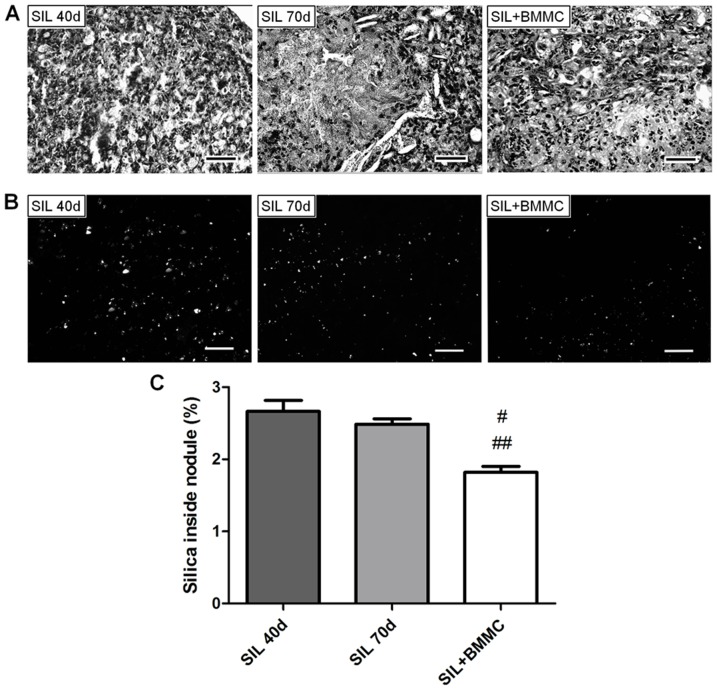
BMMC therapy reduces silica particle inside nodules. Photomicrographs of (**A**) silicotic nodules and (**B**) silica particle in nodules under polarization microscopy. Bars: 100 µm. (**C**) Quantification of silica inside nodules. Data are presented as the mean ± SEM. *n* = 7 animals per group. ^#^Significantly different from SIL40d. ^##^Significantly different from SIL70d.

Quantification of the silica particles under polarizing light showed reduced silica inside nodules in the SIL+BMMC group compared with the SIL40d and SIL70d groups (*P*<0.05) (Fig. 5).

**Figure 6:**
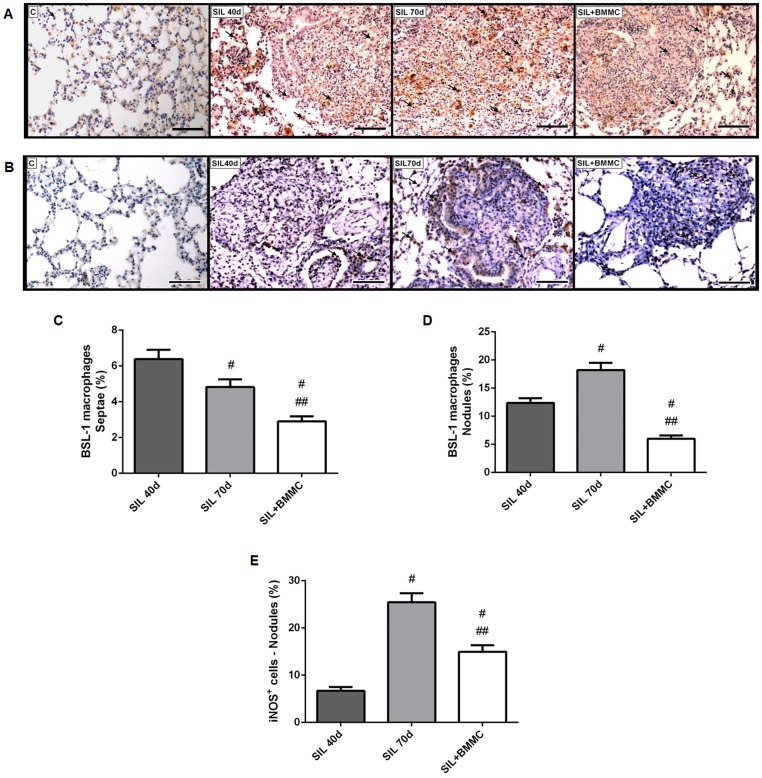
BMMC therapy decreases the presence of macrophages and cells reactive for iNOS. Photomicrographs of lung parenchyma after histochemistry with (**A**) BSL-1 lectin and (**B**) iNOS antibody. Note the macrophages and cells reactive for iNOS (arrows) in the lung tissue. Bars: 100 µm. Quantification of BSL-1 macrophages in (**C**) septae and (**D**) nodules. (**E**) Quantification of iNOS cells in nodules. Data are presented as the mean ± SEM. *n* = 7 animals per group. ^#^Significantly different from SIL40d. ^##^Significantly different from SIL70d.

BSL-1 reactive macrophages were present in some isolated alveolar spaces, in alveolar septa, and nodules in SIL40d, SIL70d, and SIL+BMMC animals (Fig. 6A). The number of BSL-1 reactive macrophages in alveolar septa was higher in SIL40d animals compared with SIL70d and SIL+BMMC animals (*P*<0.05) (Fig. 6C). SIL+BMMC animals showed the greatest reduction in BSL-1–positive macrophages among the groups (*P*<0.05) (Fig. 6C). In contrast, BSL-1 reactivity inside silica nodules was significantly higher in SIL70d animals compared with SIL40d and SIL+BMMC animals (*P*<0.05) (Fig. 5D). iNOS-reactive cells were present in nodules (Fig. 6B). They were more numerous in SIL70d nodules compared with SIL40d nodules and reduced in the SIL+BMMC group compared with the SIL70d group (*P*<0.05) (Fig. 6E). iNOS reactive cells were rarely seen inside alveolar septae (data not shown).

### TFG-β expression and apoptosis

**Figure 7:**
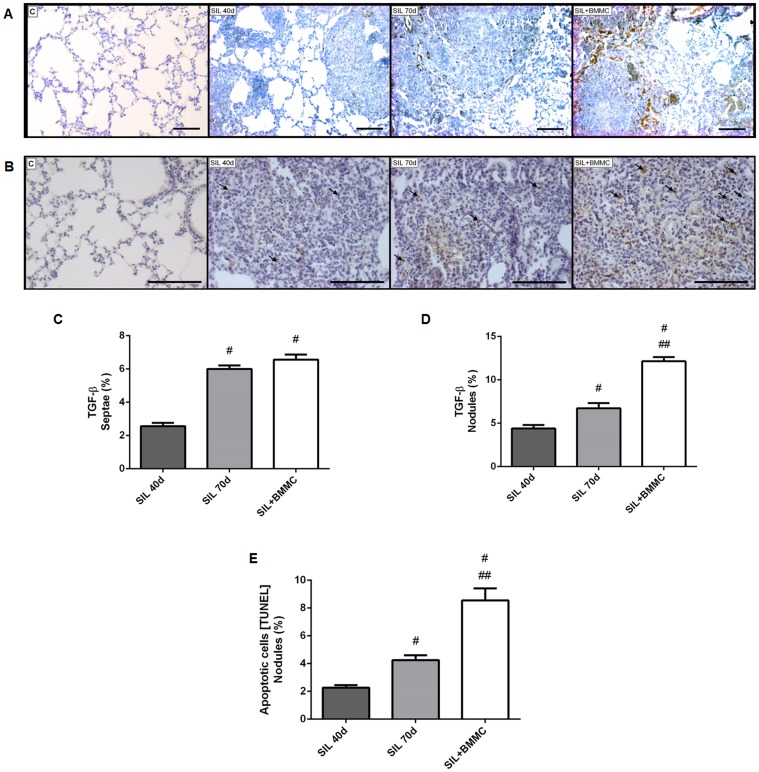
BMMC therapy increases TGF-β expression and cell apoptosis. Photomicrographs of lung parenchyma after immunohistochemistry with (**A**) TGF-β and (**B**) the TUNEL method. Note the apoptotic cells (arrows) in the silicotic nodules. Bars: 100 µm. Quantification of TGF-β in (**C**) septae and (**D**) nodules. (**E**) Quantification of apoptotic cells in nodules. Data are presented as the mean ± SEM. *n* = 7 animals per group. ^#^Significantly different from SIL40d. ^##^Significantly different from SIL70d.

TGF-β immunoexpression was predominantly seen in cells (Fig. 7A). In septae, the amount of TGF-β was significantly higher in SIL70d and SIL+BMMC animals than in SIL40d animals (*P*<0.05) (Fig. 7C). SIL70d and SIL+BMMC animals had similar amounts of TGF-β inside alveolar septae (Fig. 7C). In nodules, the amount of TGF-β was higher in SIL70d and SIL+BMMC animals compared with SIL40d animals (*P*<0.05) but the SIL+BMMC group had the highest level of TGF-β (Figs. 7D) and apoptotic cells. The percentage of apoptotic cells (apoptotic index) increased with time after silica-induced injury (Fig. 7B). The highest index occurred in SIL+BMMC animals compared with SIL40d and SIL70d animals (*P*<0.05) (Fig. 7E).

### Expansion of T regulatory cells

**Figure 8:**
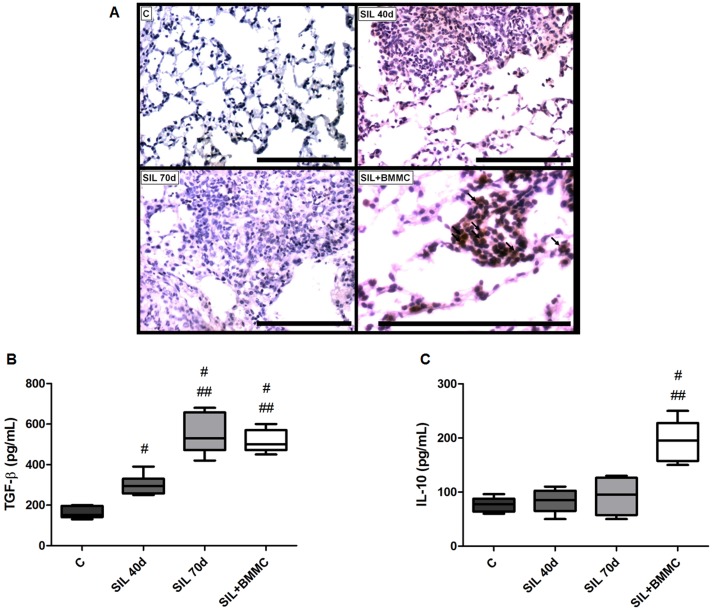
BMMC therapy leads to expansion and recruitment of T regulatory cells. Photomicrographs of lung parenchyma after immunohistochemistry with FoxP3 antibody (**A**). Note the T regulatory cells (arrows) only in the SIL+BMMC group. Bars: 100 µm. Quantification of (**B**) TGF-β and (**C**) IL-10 by enzyme-linked immunosorbent assay. Data are presented as the mean ± SEM. *n* = 7 animals per group. ^#^Significantly different from C. ^##^Significantly different from SIL 40d. ^£^Significantly different from SIL70d.

T regulatory cells were observed only in the lungs of animals in the SIL+BMMC group (Fig. 8A). Concomitant with the presence of T regulatory cells, an increase in the level of IL-10 was observed in the SIL+BMMC group compared with the C groups. The level of IL-10 in SIL40d and SIL70d animals was not different compared with C groups (Fig. 8B). Furthermore, an increase in the level of TGF-β was observed in SIL groups compared with C groups. SIL+BMMC and SIL70d animals presented a greater increased level of TGF-β compared with SIL40d animals (Fig. 8C).

### Lung fibrosis

**Figure 9:**
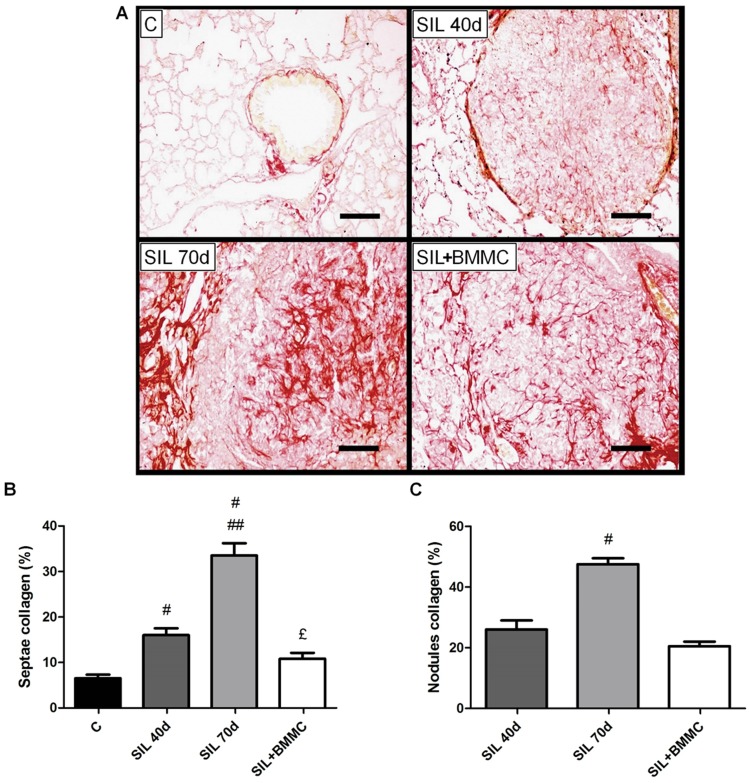
BMMC therapy reduces collagen deposition. (**A**) Photomicrographs of lung parenchyma stained with Sirius Red from animals in the C, SIL40d, SIL70d and SIL+BMMC groups. Bars: 100 µm. Quantification of collagen deposition on (**B**) septae and (**C**) nodules. Data are presented as the mean ± SEM. *n* = 7 animals per group. ^#^Significantly different from C. ^##^Significantly different from SIL40d. ^£^Significantly different from SIL70d.

The control groups presented collagen deposition around bronchi and blood vessels with minimal reactivity in alveolar septae (Fig. 9A). Collagen was evident in alveolar septae, around nodules, and inside nodules in SIL40d animals. Thin fibres were deposited in nodules in a dispersed pattern at this stage. In SIL70d animals, collagen deposits were thicker and arranged around the nodules and in the middle of the nodules (Fig. 9A). SIL+BMMC animals showed a thinner and more dispersed pattern of collagen deposition inside nodules. Histomorphometric analyses of fibrosis demonstrated that the amount of collagen increased with time in both locations, alveolar septae and nodules, and that the treatment with BMMCs led to significantly decreased alveolar septae and fibrotic nodule (*P*<0.05) (Fig. 9B,C).

## Discussion

Silicosis is a chronic fibrotic disorder that progressively leads to respiratory failure. Chronic inhalation of silica particles leads to cycles of cell activation, silica particle phagocytosis, cell death, and release of inflammatory/profibrotic mediators such as cytokines, arachidonic acid metabolites (eicosanoids), ROS and reactive nitrogen species (RNS). These mediators induce pronounced recruitment of inflammatory cells both in the alveolar walls and alveolar spaces [26], dominated by alveolar macrophages, but also including neutrophils and lymphocytes [27].

No available curative therapy exists for silicosis [6], therefore cell therapy based on stem cell infusion seems to be a sensible approach. Several studies investigating the therapeutic role of these cells have used specific subpopulations, such as hematopoietic stem cells, the mononuclear fraction (BMMCs), and mesenchymal stromal cells. BMMCs are of particular interest, mainly because of ethical considerations for medical application but also because they are easily harvested, isolated and purified. Previous studies have demonstrated that infusion of BMMCs 1 h after induction of silicosis in mice led to improvement in lung inflammation and fibrosis in lesions after 15 days [18]. These results are quite similar to other disease models in which the infusion of BMMCs by the systemic route at the time of injury leads to attenuation of the lesion. The beneficial outcome seems to be due to anti-inflammatory, anti-apoptotic, and anti-fibrotic effects [18], [28], [29], sometimes associated with functional improvement and a decrease in mortality [10], [27]. Lassance et al. [19] treated 15-day silica-damaged mice with bone marrow-derived cells by the intratracheal route. Their results show that 15 days later, lung function and histological parameters such as granuloma area fraction and inflammation improved. However, these beneficial effects reverted to the silica-injured levels 45 days after infusion of bone marrow-derived cells [19]. Our morphological approach highlights some clues about the mechanisms involved in the apparent improvement in the disease. Even when BMMCs were infused at 40 days, the results for the silica-injured lungs show that the functional parameters of the lungs were reversed (Fig. 2). Moreover, the functional changes could be related to the intense decrease in collagen deposition (Fig. 9) as well as to the significant decrease in the total area occupied by small nodules (<100,000) in SIL+BMMC animals compared with SIL70d animals (318.20 vs 370.77; *P*<0.05). The diminution in collagen deposition could be explained by the increase in secretion/activation of metalloproteinases, enzymes capable of degrading the extracellular matrix. This has been already demonstrated in a model of cirrhosis induced by CCl_4_ [30], [31]. In CCl_4_-damaged liver, besides an important contribution of bone marrow-derived cells as the source of metalloproteinases (MMP-13, and -9) [30], inflammatory cells and intrinsic tissue cells contribute to the release of MMPs and matrix remodelling [31]. In our study, because 99mTc-BMMCs did not homing in lung tissue until 24 h after of injection and chromosome Y from donor cells was not detected in silica-damaged lungs 30 days after treatment (Fig. 1), MMPs might be mainly derived from inflammatory cells. Moreover, the decrease in fibrosis could also be explained by downregulation of the tissue inhibitor of MMPs provoked by the infusion of BMMCs, which could also favour the degradation of fibrosis [32]. Several lines of evidence show that the infusion of bone marrow stem cells, in particular mesenchymal stem cells, induce an anti-inflammatory response [5], [7], [12], [14]. However, inflammation was not decreased after infusion of BMMCs in our study (Fig. 4). The cell counts in BALF and in nodules reveal an increase in cellularity after infusion of BMMCs (Fig. 3), but the number of BSL-1 reactive macrophages diminished (Fig. 6). The lymphocyte aggregates present in nodules might have given rise to the increased cell count. This lymphocyte population could originate from the expanded T regulatory cells, which have a crucial role in the maintenance of immune homeostasis in the airways. It has been shown that, in silica-damaged lungs, T regulatory cells maintain immune homeostasis through IL-10 and/or TGF-β secretion [33], [34] and through some other inhibitory molecules including CTLA-4 [35]. At the early stage of silicosis, T regulatory cells mainly inhibited inflammation by CTLA-4 molecules. With the development of silicosis, T regulatory cells suppress the Th1 immune response by secreting increasing amounts of IL-10 and TGF-β. Then, Th1/Th2 polarization shifts towards a Th2-dominant immune response [36]. The importance of T regulatory cells in modulating inflammation is evident after the administration of mesenchymal stromal cells both in the mouse model of colitis induced by trinitrobenzene sulfonic acid [37] and in patients with fistulizing Crohn disease [38]. This strategy led to an improvement in the disease and an increase in circulating FoxP3^+^ regulatory T cells. Furthermore, it was previously demonstrated that the increase amount of T reg cells cause an increase in MMP activity [39], [40]. Our results showed a massive presence of FoxP3^+^ cells and an increased level of IL-10 and TGF-β in the SIL+BMMC group (Fig. 8). This growth factor act as an autoregulatory loop inducing the expansion of T regulatory cells, which in turn secrete TGF-β1 [41], which is critical for the development and differentiation of FoxP3^+^ regulatory T cells [41]. Moreover, TGF-β1 has a potent effect on inhibition of the immune response, particularly T cell proliferation and differentiation [42], [43], which corroborates the anti-inflammatory effect of bone marrow-derived cells [44], [45]. An anti-fibrotic effect is also demonstrated through the induction of Smad-mediated IL-10 secretion, a co-secretion responsible for the control of fibrosis [46].

In response to silica, alveolar macrophages upregulate both IL-1β and iNOS gene expression [47], [48]. IL-1β and NO mediate apoptosis and inflammation in murine silicosis [49]. The decrease in the number of cells iNOS immunoreactivity after infusion of BMMCs demonstrated in this work (Fig. 6), and consequently NO synthesis, could not be responsible for the increased apoptosis, reinforcing the role of TGF-β in the mediation of apoptosis (Fig. 7). Furthermore, the decrease in iNOS favours a cytoprotective role for the infusion of BMMCs because the sites of iNOS activation and NO-mediated damage are associated temporally and anatomically with lesions in the lung [48]. However, the increase of LDH secretion and maintenance of MPO activity in SIL+BMMC group compared to SIL group in the lung parenchyma suggests the persistence of remodelling of the nodules with will lead decrease of further inflammatory cell population in the lung. It is possible that this macrophage activation is related to the uptake of silica and subsequent translocation of silica to alveolar interstitial spaces and its drainage to lymph nodes, which could explain the diminished amount of silica seen inside nodules after infusion of BMMCs.

In conclusion, the infusion of BMMCs in the late stages of silica-induced damage was able to induce an improvement in lung function, mainly due to an important decrease in fibrosis. However, the morphological data show that the inflammatory process was maintained with a change in the profile of the cells; a subset of the T regulatory cell population may possibly have been recruited to exert an anti-fibrotic and protective role.
